# Urinary albumin/creatinine ratio tertiles predict risk of diabetic retinopathy progression: a natural history study from the Adolescent Cardio-Renal Intervention Trial (AdDIT) observational cohort

**DOI:** 10.1007/s00125-022-05661-1

**Published:** 2022-02-19

**Authors:** Paul Z. Benitez-Aguirre, M. Loredana Marcovecchio, Scott T. Chiesa, Maria E. Craig, Tien Y. Wong, Elizabeth A. Davis, Andrew Cotterill, Jenny J. Couper, Fergus J. Cameron, Farid H. Mahmud, H. Andrew W. Neil, Timothy W. Jones, Lauren A. B. Hodgson, R. Neil Dalton, Sally M. Marshall, John Deanfield, David B. Dunger, Kim C. Donaghue, on behalf of the Adolescent Type 1 Diabetes Cardio-Renal Intervention Trial (AdDIT)

**Affiliations:** 1 Institute of Endocrinology and Diabetes, The Children’s Hospital at Westmead, Sydney, Australia; 2 Discipline of Child and Adolescent Health, University of Sydney, Sydney, NSW Australia; 3 Department of Paediatrics, University of Cambridge, Cambridge, UK; 4 Institute of Cardiovascular Science, University College London, London, UK; 5 School of Women’s and Children’s Health, University of New South Wales, Sydney, NSW Australia; 6 Centre for Eye Research Australia, Melbourne, VIC Australia; 7 Singapore Eye Research Institute, Singapore National Eye Centre, Singapore, Singapore; 8 Duke-NUS Medical School, National University of Singapore, Singapore, Singapore; 9 Department of Endocrinology and Diabetes, Princess Margaret Hospital for Children, Perth, WA Australia; 10 Telethon Kids Institute, University of Western Australia, Perth, WA Australia; 11 University of Queensland, Brisbane, QLD Australia; 12 Endocrinology and Diabetes Centre, Women’s and Children’s Hospital, and Robinson Institute, University of Adelaide, Adelaide, SA Australia; 13 Department of Endocrinology and Diabetes, Royal Children’s Hospital, Melbourne, VIC Australia; 14 Murdoch Children’s Research Institute, Melbourne, VIC Australia; 15 The University of Melbourne, Melbourne, VIC Australia; 16 Division of Endocrinology, Hospital for Sick Children, Toronto, ON Canada; 17 Oxford Centre for Diabetes, Endocrinology and Metabolism, University of Oxford, Oxford, UK; 18 St Thomas’ Hospital, Well Child Laboratory, Evelina London Children’s Hospital, London, UK; 19 Translational and Clinical Research Institute, Newcastle University, Newcastle, UK; 20 Institute of Metabolic Science, University of Cambridge, Cambridge, UK

**Keywords:** AdDIT, Adolescents, Diabetic nephropathy, Diabetic retinopathy progression, Kidney function, Microvascular complications, Type 1 diabetes

## Abstract

**Aims/hypothesis:**

We hypothesised that adolescents with type 1 diabetes with a urinary albumin/creatinine ratio (ACR) in the upper tertile of the normal range (high ACR) are at greater risk of three-step diabetic retinopathy progression (3DR) independent of glycaemic control.

**Methods:**

This was a prospective observational study in 710 normoalbuminuric adolescents with type 1 diabetes from the non-intervention cohorts of the Adolescent Cardio-Renal Intervention Trial (AdDIT). Participants were classified as ‘high ACR’ or ‘low ACR’ (lowest and middle ACR tertiles) using baseline standardised log_10_ ACR. The primary outcome, 3DR, was determined from centrally graded, standardised two-field retinal photographs. 3DR risk was determined using multivariable Cox regression for the effect of high ACR, with HbA_1c_, BP, LDL-cholesterol and BMI as covariates; diabetes duration was the time-dependent variable.

**Results:**

At baseline mean ± SD age was 14.3 ± 1.6 years and mean ± SD diabetes duration was 7.2 ± 3.3 years. After a median of 3.2 years, 83/710 (12%) had developed 3DR. In multivariable analysis, high ACR (HR 2.1 [1.3, 3.3], *p*=0.001), higher mean IFCC HbA_1c_ (HR 1.03 [1.01, 1.04], *p=*0.001) and higher baseline diastolic BP SD score (HR 1.43 [1.08, 1.89], *p*=0.01) were independently associated with 3DR risk.

**Conclusions/interpretation:**

High ACR is associated with greater risk of 3DR in adolescents, providing a target for future intervention studies.

**Trial registration:**

isrctn.org ISRCTN91419926.

**Graphical abstract:**

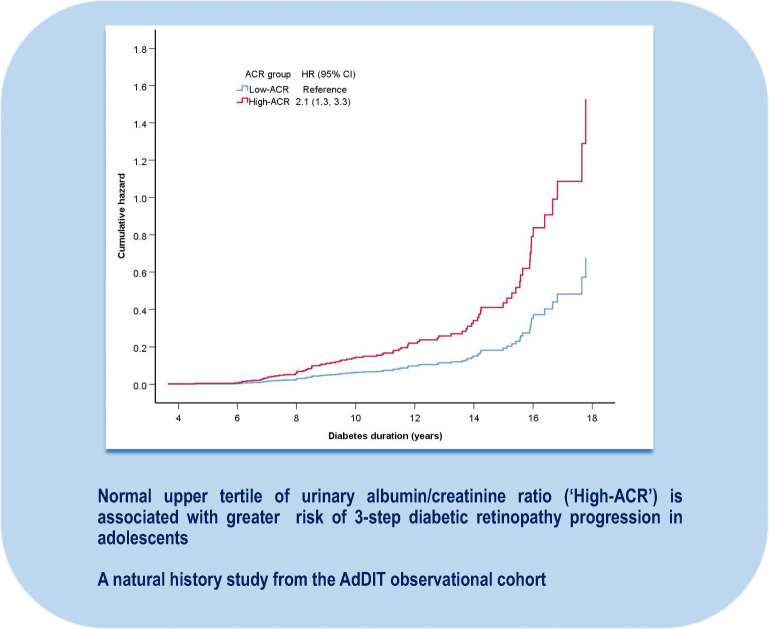

**Supplementary Information:**

The online version contains peer-reviewed but unedited supplementary material available at 10.1007/s00125-022-05661-1.

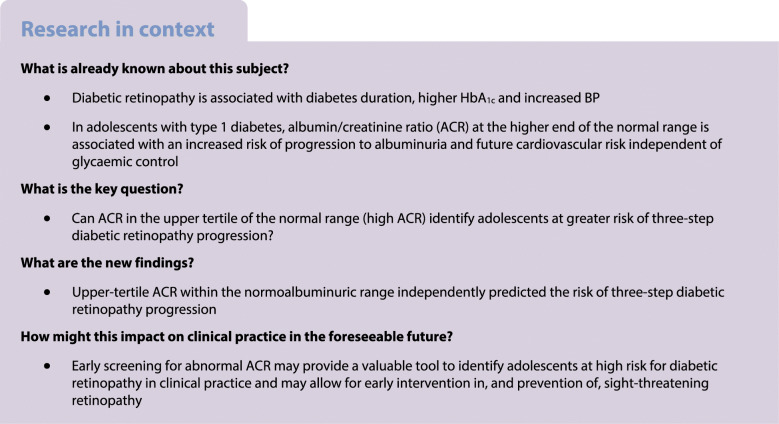



## Introduction

Prevention of sight-threatening diabetic retinopathy through early intervention requires timely screening and identification of people at greatest risk of diabetic retinopathy progression [1]. Urinary albumin/creatinine ratio (ACR) within the upper tertile (high ACR) of the normoalbuminuric range during the early years following type 1 diabetes diagnosis is associated with future risk of kidney disease [2] and cardiovascular risk [3], impaired cardiac autonomic function [4] and early alterations in the retinal microvasculature [5], when compared with a lower ACR despite shorter diabetes duration. However, an association between ACR and risk of diabetic retinopathy progression has not been clearly established in youth with type 1 diabetes. Glycaemic control and diabetes duration are the most consistently shown determinants for diabetic retinopathy progression [1].

In addition to active intervention, the Adolescent Type 1 Diabetes Cardio-Renal Intervention Trial (AdDIT) included a parallel observational (non-intervention) natural history cohort of participants with high ACR and low ACR in whom the outcome of diabetic retinopathy was examined. Utilising this observational cohort, we hypothesised that high ACR is associated with greater risk of diabetic retinopathy progression independent of glycaemic control.

## Methods

### Study population

Overall, 4407 adolescents with type 1 diabetes were screened for participation in AdDIT, each providing three consecutive early morning urine samples at two separate visits. Centralised assessment of all urine samples was performed at the WellChild Laboratory, Evelina Children’s Hospital, London. The average residual was calculated using age, sex and duration and the coefficients from the previous models [6]. ACR tertile assignment was as follows: upper-tertile (high ACR group) ACR >1.2 middle-tertile ACR 0.8–1.2 and lower-tertile ACR <0.8. The lower two tertiles were combined for analysis as the ‘low ACR’ group [7].

We assessed 710 natural history participants (510 low ACR and 200 high ACR) who attended repeat annual standardised visits and had gradable retinal photography across three countries (UK, Canada and Australia) using protocols previously described [5]. Anonymised digital retinal photographs were centralised to the Centre for Eye Research Australia, Melbourne, VIC, Australia for diabetic retinopathy grading according to the Early Treatment Diabetic Retinopathy Study [8] by expert graders masked to ACR tertile and clinical characteristics. Three-or-more-step diabetic retinopathy progression (3DR) in the worse eye was the primary outcome measure, as used in the DCCT [9]; the minimum grade of those with 3DR was grade 31.

HbA_1c_ was analysed at each centre, using DCCT-aligned methods [7]. HbA_1c_ results were retrieved from clinical databases to calculate mean HbA_1c_ values through the study period. Upper HbA_1c_ tertile was assigned to mean HbA_1c_ values ≥74 mmol/mol (8.9%) and compared with the lower two HbA_1c_ tertiles combined into a single category (HbA_1c_ ≥74 mmol/mol [8.9%] vs HbA_1c_ <74 mmol/mol [8.9%]). Lipid profile (cholesterol, HDL-cholesterol, LDL-cholesterol, triacylglycerols) was measured using routine laboratory methods [7].

Height, weight and BMI SD scores (SDSs) were calculated according to the least mean squares method [10]. BP was measured (mean of two measures) using an Omron M6 BP (all centres) with an appropriately sized cuff with SDS calculated [11]. The study was approved by the Cambridge University Hospitals Research Ethics Committee and local ethics committees internationally. Parents and participants provided written informed consent and assent.

### Statistics

Descriptive baseline statistics comparing high vs low ACR and 3DR progressors vs 3DR non-progressors are presented as mean ± SD for normally distributed data, median (IQR) for skewed distributions and as *n* (%) for proportions. Differences between continuous independent samples were evaluated using independent *t* tests for normally distributed data, or Kruskal–Wallis test for skewed data. χ^2^ test was used to determine differences between proportions.

The primary outcome measure was 3DR, which was examined using Cox proportional hazard regression. Diabetes duration was used as the time-dependent variable. HRs and 95% CIs are reported per one unit change in the risk factor. Explanatory variables included the following: high ACR and low ACR; mean HbA_1c_ and HbA_1c_ ≥74 mmol/mol (8.9%); BP SDS; BMI SDS; and LDL-cholesterol and diabetic retinopathy status at baseline. All statistical analyses were conducted using SPSS version 25 (https://www.ibm.com/au-en/products/spss-statistics).

## Results

At baseline, mean ± SD age was 14.3 ± 1.6 years and mean ± SD diabetes duration was 7.2 ± 3.3 years. There were no significant differences between the high ACR and low ACR groups with respect to age, sex distribution, systolic BP (SBP) SDS, diastolic BP (DBP) SDS, BMI SDS, HbA_1c_ or LDL-cholesterol. The high ACR group had shorter diabetes duration (electronic supplementary material [ESM] Table 1). Participants had a median (IQR) of 4 (2–5) assessments after a median 3.2 years of follow-up; 3DR developed in 83/712 (11.7%). Cumulative incidence of 3DR in the high vs low ACR group was 15.5% vs 10.2%, *p*=0.048 (ESM Table 1).

In univariable Cox regression analysis, high ACR, higher HbA_1c_ and higher DBP SDS were associated with greater risk of 3DR (Table 1).

**Table 1:** Risk of 3DR

Characteristic	Univariable model		Multivariable model	
	HR (95% CI)	*p* value	HR (95% CI)	*p* value
High ACR	2.3 (1.4, 3.5)	0. 001	2.1 (1.3, 3.3)	0.001
Female sex	1.2 (0.8, 1.8)	0.5	–	–
Mean HbA_1c_ (mmol/mol)	1.03 (1.02, 1.05)	<0.0001	1.03 (1.01, 1.04)	0.001
Mean HbA_1c_ (%)	1.40 (1.19, 1.65)	<0.0001		
Baseline HbA_1c_ (mmol/mol)	1.03 (1.01, 1.04)	<0.0001		
Baseline HbA_1c_ (%)	1.31 (1.12, 1.54)	0.001		
Baseline SBP (mmHg)	1.01 (1.00, 1.03)	0.1	–	–
Baseline DBP (mmHg)	1.04 (1.01, 1.06)	0.006	–	–
Baseline SBP SDS	1.21 (0.97, 1.50)	0.1		
Baseline DBP SDS	1.48 (1.12, 1.95)	0.006	1.43 (1.08, 1.89)	0.01
Baseline BMI SDS	1.25 (0.96, 1.62)	0.1	–	–
Baseline LDL-cholesterol (mmol/l)	1.13 (0.87, 1.46)	0.4	–	–
Baseline LDL >2.6 mmol/l	1.24 (0.79, 1.96)	0.4	–	–
Retinopathy at baseline	0.92 (0.53, 1.76)	0.9	–	–

Cox regression analysis with diabetes duration as time-dependent variable

**Fig. 1:**
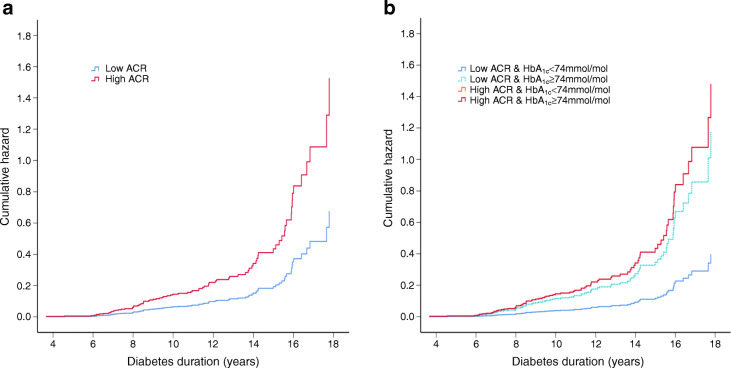
Cox regression analysis of high vs low ACR for risk of 3DR. (**a**) Risk of 3DR by ACR group and model adjusted for mean HbA_1c_ and DBP SDS. High ACR vs low ACR (HR 2.1 [1.3, 3.3]). (**b**) Risk by ACR and HbA_1c_ ≥74 mmol/mol (8.9%) model adjusted for DBP SDS. Upper-tertile ACR (high ACR) is associated with risk of 3DR. Glycaemic control modifies risk of 3DR particularly in the low ACR group. HR (95% CI): Low ACR & HbA_1c_ <74 mmol/mol, 1.0 (reference); Low ACR & HbA_1c_ ≥74 mmol/mol, 3.0 (1.7, 5.1); High ACR & HbA_1c_ <74 mmol/mol, 3.7 (1.9, 7.1); High ACR & HbA_1c_ ≥74 mmol/mol, 3.7 (1.9, 7.2) The orange line (High ACR & HbA1c <74mmol/mol) is not visible because it is obscured by the red line (High ACR & HbA1c ≥74 mmol/mol), due to similar HR

In multivariable Cox regression analyses, greater 3DR risk was associated with high ACR (HR 2.1 [1.3, 3.3], *p*=0.001), IFCC HbA_1c_ (HR 1.03 [1.01, 1.04], *p*=0.001) and DBP SDS (HR 1.43 [1.08, 1.89], *p*=0.01) (Fig. 1). 3DR risk was not associated with diabetic retinopathy at baseline, nor lipid levels nor BMI (Table 1).

In the low ACR group, HbA_1c_ ≥74 mmol/mol (8.9%) significantly increased 3DR risk to that comparable with the high ACR groups. In the high ACR groups, HbA_1c_ ≥74 mmol/mol was not associated with greater 3DR risk (Fig. 1).

## Discussion

Previously reported data from the AdDIT cohorts highlighted the systemic nature of the pre-clinical diabetic endotheliopathy by describing that high ACR was associated with changes in retinal vascular geometry [5], greater risk of albuminuria and greater thickening of carotid intima–media thickness [12]. In this multinational AdDIT natural history cohort, we demonstrate that upper-tertile ACR (high ACR group) within the normoalbuminuric range was associated with greater risk of 3DR after adjusting for HbA_1c_. Furthermore, we demonstrate that early rise in DBP and HbA_1c_ ≥74 mmol/mol (8.9%) significantly increased risk of 3DR particularly in the low ACR group.

Interestingly, in the high ACR group, higher mean HbA_1c_ (≥74 mmol/mol [8.9%]) did not significantly modify risk of 3DR, suggesting that the inherent biological risk for progression of microvascular complications may be largely independent of appropriate glycaemic control. This is important in clinical care settings, as individuals identified as ‘high risk’ through ACR screening should continue to be closely monitored for complications despite optimal glycaemic control and highlights a need for interventions other than glycaemic control to ameliorate risk and progression diabetic retinopathy.

Our findings of greater risk in the high ACR group complement the diabetic retinopathy screening advice for adolescents arising from the DCCT/EDIC [13] study group, primarily based on HbA_1c_ levels. At the same time, our findings are in keeping with the greatest risk factors for proliferative diabetic retinopathy in the DCCT, including an elevated urinary albumin excretion rate and higher mean DBP [14]. Importantly, in our study, the presence or absence of diabetic retinopathy at baseline did not influence risk of 3DR, thus further demonstrating the robust nature of stratification by ACR groups in youth with shorter diabetes duration. Notably, the high ACR group had a lower proportion of diabetic retinopathy at baseline, likely related to shorter diabetes duration. In keeping with our hypothesis, a higher proportion of high ACR participants developed 3DR with ongoing diabetes exposure despite shorter diabetes duration. Those with high ACR appear to have an underlying predisposition for a systemic endotheliopathy that progresses more rapidly as evidenced by 3DR. The evidence supports both genetic and metabolic mechanisms that protect and predispose from diabetes complications [15], although clinically measurable and reproducible biomarkers associated with such risk have been elusive. Our data suggest that broader screening through ACR may assist to identify a ‘high risk’ group in the population who are predisposed to earlier onset of complications and likely to benefit from earlier intervention.

In the low ACR group, those with HbA_1c_ ≥74 mmol/mol (8.9%) had significantly increased risk of 3DR similar to the high ACR group, thus confirming that HbA_1c_ significantly influences and modifies diabetic retinopathy and in keeping with findings from the DCCT/EDIC studies [16]. Hence, screening for microvascular complications is influenced by an inherent biological predisposition, which is significantly modified by glycaemic exposure.

In addition, an early elevation of DBP even within the normotensive range significantly increased risk of 3DR, consistent with our previous findings that DBP and SBP increases within the normotensive range associate with incident diabetic retinopathy in adolescents with type 1 diabetes [17].

The strengths of our study include a large multinational population from a study collaboration with standardised methods. Limitations include a low number of photographs, relatively short time in study period and the post hoc examination of these cohorts. However, we analysed a non-intervention population and used total diabetes duration as our time-dependent variable since the predominant effect of duration is more pronounced for diabetic retinopathy. Furthermore, the low ACR group had longer diabetes duration, thereby making an underestimate of 3DR unlikely in this group compared with the high ACR group.

In conclusion, we demonstrate that urinary ‘high ACR’, albeit in the normoalbuminuria range, identifies adolescents at greater risk of diabetic retinopathy progression. This was despite shorter diabetes duration and after adjusting for glycaemic exposure. We also observed that early DBP elevation significantly modifies 3DR risk. Higher glycaemic burden increases risk of 3DR particularly in the low ACR group and remains a crucial target for intervention. Further research to translate the ACR screening threshold into real-world application is required. The longitudinal follow-up from AdDIT cohorts will provide invaluable insight into the mechanisms underlying diabetes complications and the potential benefits of early ‘pre-complications’ interventions in paediatric cohorts.

### Supplementary Information

ESM (PDF 34 kb)

## Data Availability

The datasets generated during and/or analysed during the current study are available from the AdDIT Steering Committee through the corresponding author on reasonable request. Data repository name: Department of Paediatrics, University of Cambridge.

## Appendix

**AdDIT investigators** Australia: Tim Jones (Perth); Kim Donaghue (Sydney); Maria Craig (Sydney); Fergus Cameron (Melbourne); Jennifer Couper (Adelaide); Elizabeth Davis (Perth); Andrew Cotterill (Brisbane); Bruce King (Newcastle); Charles Verge (Sydney); Phil Bergman (Victoria); Christine Rodda (Victoria); and Paul Benitez-Aguirre (Sydney). UK: Carlo Acerini (Cambridge)^a^; Fran Ackland (Northampton); Binu Anand (West Suffolk); Tim Barrett (Birmingham); Virginia Birrell (Middlesbrough); Fiona Campbell (Leeds);Tim Cheetham (Newcastle upon Tyne); Chris Cooper (Stockport); Ian Doughty (Manchester); Atanu Dutta (Stoke Mandeville); Julie Edge (Oxford); Julian Hamilton-Shield (Bristol); James Heywood (Cambridge); Nicola Leech (Newcastle upon Tyne); Nick Mann (Reading); Richard Parker (Cambridge); Gerry Rayman (Ipswich); Jonathon Mark Robinson (Wigan); Michelle Russell-Taylor (High Wycombe); Vengudi Sankar (Bolton); Nandu Thalange (Norwich); and Mark Wilson (Cambridge). Canada: Farid Mahmud (Toronto); Cheril Clarson (London, Ontario); Jacqueline Curtis (Toronto); Etienne Sochett (Toronto); and Denis Daneman (Toronto).

^a^Carlo Acerini died in May 2019, prior to publication of this work.

## Abbreviations

3DR: Three-or-more-step diabetic retinopathy progression
ACR: Albumin/creatinine ratio
AdDIT: Adolescent Type 1 Diabetes Cardio-Renal Intervention Trial
DBP: Diastolic BP
SBP: Systolic BP
SDS: SD score
