# Supplementary material for: Urinary albumin/creatinine ratio tertiles predict risk of diabetic retinopathy progression: a natural history study from the Adolescent Cardio-Renal Intervention Trial (AdDIT) observational cohort
Source: Diabetologia. 2022 Feb 19;65(5):872–8. doi: 10.1007/s00125-022-05661-1 (PMC8960571; doi:10.1007/s00125-022-05661-1)
Supplement: Supplementary file 1 — (PDF 34 kb) [file 125_2022_5661_MOESM1_ESM.pdf]

	**High-ACR (n=200)**	**Low-ACR (n=510)**	**P**	**3DR (n=83)**	**No 3DR (n=627)**	**p**
High ACR n (%)	---	---	--	31 (37.3%)	170 (27.1%)	0.05
Retinopathy at Baseline	12/197 (6.1)	96/501 (19.2)	<0.0001	11/82 (13.4)	97/616 (15.7)	0.6
3DR Cumulative incidence (%)	15.5	10.2	0.048	--	--	--
Baseline Age (years)	14.3 (1.5)	14.3 (1.6)	0.7	14.8 [13.8, 15.9]	14.4 [13.0, 15.6]	0.02
Age at diagnosis (years)	8.2 (3.1)	6.7 (3.4)	<0.0001	6.6 (3.4)	7.2 (3.4)	0.1
Age at Final visit (years)	17.5 (1.6)	17.3 (1.8)	0.052	17.5 [16.5, 18.7]	17.5 [16.1, 18.6]	0.4
Duration at Baseline Visit (years)	5.5[3.7, 8.2]	7.4 [5.0, 10.1]	<0.0001	7.4 [5.2, 10.1]	6.6 [4.3, 9.2]	0.01
Duration at Final Visit (years)	8.9 [6.9, 11.5]	10.4 [7.8, 13.1]	<0.0001	11.6 [9.0, 15.0]	10.2 [8.0, 13.3]	0.004
Time in Study (years)	3.3 [2.7, 4.0]	3.1[2.1, 3.9]	<0.0001	3.0 [2.0, 3.9]	3.2 [2.2, 3.9]	0.08
Baseline HbA1c *(mmol/mol)*	69.4 (15.3)	68.3 (14.2)	0.3	73.8 (15.3)	68.3 (14.2)	0.001
Baseline HbA1c (%)	8.5 (1.4)	8.4 (1.3)	0.3	8.9 (1.4)	8.4 (1.3)	0.001
Mean Study HbA1c *(mmol/mol)*	71.6 (14.2)	70.5 (14.1)	0.2	77.0(15.3)	69.4 (14.1)	<0.0001
Mean Study HbA1c (%)	8.7 (1.3)	8.6 (1.2)	0.2	9.2 (1.4)	8.5 (1.2)	<0.0001
Baseline SBP (mmHg)	116 [108, 121]	114[108, 123]	0.8	118 [109, 124]	114 [108, 122]	0.1
Baseline SBP SDS	0.56 (0.98)	0.51 (0.91)	0.5	0.64 (0.93)	0.51 (0.93)	0.2
Baseline DBP (mmHg)	65 [60, 72]	65 [60-71]	0.8	68 [62, 75]	65 [60, 71]	0.001
Baseline DBP SDS	0.80 (0.04)	0.33 (0.80)	0.7	0.59 (0.72)	0.29 (0.79)	0.001
Baseline BMI (kg/m^2)	21.8 (3.8)	22.3 (3.8)	0.1	23.1 (4.2)	22.1 (3.8)	0.04
Baseline BMI SDS	0.52 (0.86)	0.66 (0.81)	0.05	0.74 (0.82)	0.61 (0.83)	0.2
LDL-cholesterol >2.6mmol/l	17.9%	21.5%	0.5	39.4%	33.9%	0.4

ESM Table 1. Baseline Characteristics by ACR groups or 3-Step Diabetic Retinopathy (3DR) Progression Groups Variables presented as mean (SD) for parametric or median [IQR] for non-parametric variables. Proportions presented as n(%).
